# African animal trypanosomiasis as a constraint to livestock health and production in Karamoja region: a detailed qualitative and quantitative assessment

**DOI:** 10.1186/s12917-017-1285-z

**Published:** 2017-11-25

**Authors:** Dennis Muhanguzi, Albert Mugenyi, Godfrey Bigirwa, Maureen Kamusiime, Ann Kitibwa, Grace Gloria Akurut, Sylvester Ochwo, Wilson Amanyire, Samuel George Okech, Jan Hattendorf, Robert Tweyongyere

**Affiliations:** 1 College of Veterinary Medicine Animal Resources and Biosecurity, Makerere University, P.O. Box 7062, Kampala, Uganda; 2 Coordinating Office for Control of Trypanosomiasis in Uganda, Ministry of Agriculture, Animal Industry and Fisheries, Plot 78, Buganda Road, P. O. Box: 16345 Wandegeya, Kampala, Uganda; 3 Mercy Corps Uganda, PO Box 32021, Clock Tower, Kampala, Uganda; 4 Swiss Tropical Institute, Socinstrasse 57, -4002 Basel, CH Switzerland; 5 University of Basel, Petersplatz 1, 4003 Basel, Switzerland

**Keywords:** African animal trypanosomiasis, Tick-borne diseases, Control, ITS1-PCR, Prevalence, Karamoja region

## Abstract

**Background:**

Nagana (African Animal Trypanosomiasis-AAT) and tick-borne diseases (TBDs) constrain livestock production in most parts of sub-Saharan Africa. To this realisation, Uganda government set up an African trypanosomiasis (AT) control unit, which among other activities generates national tsetse control priority maps using apparent tsetse density data. Such maps underestimate mechanically transmitted AAT and thus ought to be refined using actual AT prevalence data. We therefore set out to generate up-to-date cattle and donkey trypanosomiasis prevalence data as well as find out the constraints to livestock production in Karamoja region in a bid to re-define AT control priority in this region.

**Results:**

Livestock keepers and animal health workers indicated that TBDs and AAT were the most important livestock diseases in Karamoja region. The prevalence of *Trypanosoma* spp. in cattle and donkeys was 16.3% (95% CI: 12.4–21.1%) and 32.4% (95% CI; 20.2–47.6%) respectively. *Trypanosoma vivax* (12.1%) and *Trypanosoma congolense* savannah (29.6%) were the most prevalent *Trypanosoma* spp. in cattle and donkeys respectively. Majority of the cattle (85.7%) and more than half of the donkey (57.1%) herds were positive for *Trypanosoma* spp.

**Conclusions:**

African animal trypanosomiasis and TBDs are the most important constraints to livestock production in Karamoja region. In order to improve livestock production and hence Karamajong livelihoods, government of Uganda and her development partners will need to invest in livestock health programs particularly targeting tsetse and TBD control.

## Background

African trypanosomiasis (AT) constrains livestock production and human health in 37 sub-Saharan African countries [1–3]. In these areas, about 60 million people are at moderate to high risk of acquiring sleeping sickness (human African trypanosomiasis HAT). Sleeping sickness is caused by *Trypanosoma brucei gambiense* (chronic form) and *Trypanosoma brucei rhodesiense* (acute form) [4, 5]. Both nagana and sleeping sickness are transmitted by 30 species of tsetse flies (Diptera: Glossinidae) which inhabit about 10 million km^2^ of land in the humid regions of Africa [6]. This reduces the livestock production potential for such an expanse of land [7].

**Fig. 1:**
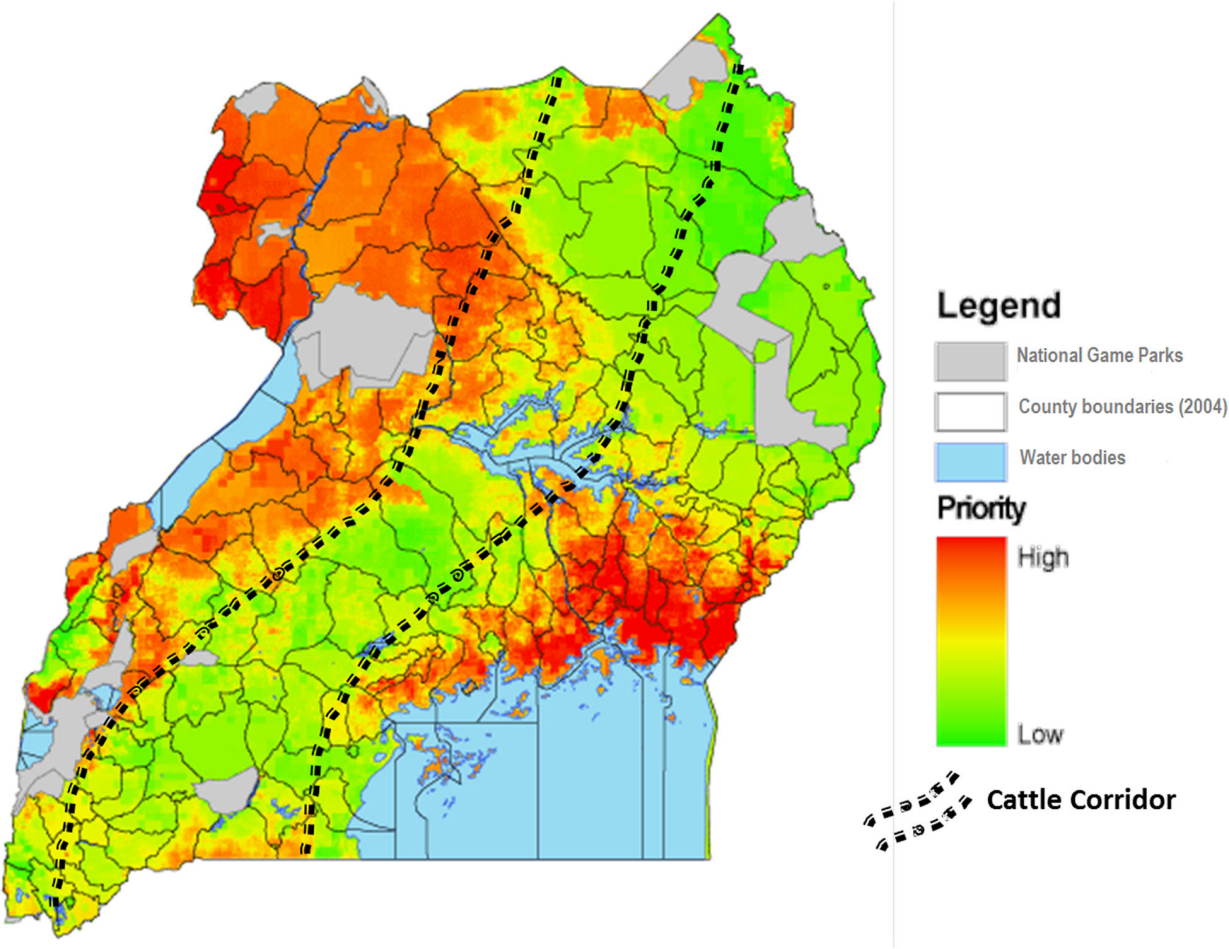
Current Uganda trypanosomiasis control priority map (Credit: Coordinating Office for Control of Trypanosomiasis in Uganda)

Tsetse and AT are considered a major livestock production [8–10] and public health constraint in Uganda [11]. For this reason, the Coordinating Office for Control of Trypanosomiasis in Uganda (COCTU), was set up to coordinate tsetse and trypanosomiasis control [12]. As part of her mandate, COCTU produces and avails tsetse and trypanosomiasis control priority maps (e.g. Fig. 1) so as to heighten vector and disease control advocacy [13]. These African trypanosomiasis (AT) control priority maps are generated from apparent tsetse density, socio-economic and environmental variables and not from actual AT prevalence data. This approach underestimates the extent of AT. This partly explains why the cattle corridor is endemic for AAT yet it is currently given low to medium priority for tsetse and AT control (Fig. 1). As such, a recent aggregate of AAT prevalence data for the East African region including Uganda presents Karamoja region with no AAT prevalence data and presumably no risk of the disease [14].

Several biting flies including tabanids can mechanically transmit *T. vivax* [15, 16]*.* As a result, *T. vivax* distribution often extends beyond tsetse belts [17, 18]. This indicates that tsetse distribution patterns cannot accurately predict the distribution of *T. vivax* AAT [15]. In addition, AT spatial distribution cannot be sufficiently predicted by apparent tsetse density (tsetse flies caught per trap per day) alone [19, 20]. The level of challenge (product of apparent tsetse density, mean tsetse fly infection rate and the proportion of feeds taken by these tsetse from livestock) is a better predictor of AT distribution [19, 20]. However, the level of challenge is often not used to predict AT distribution and to determine the level (no priority, low, moderate or high priority, etcetera) of control priority for different parts of the country. As a result, farmers in low to medium AT control priority areas including the Karamoja region have indicated that AAT is one of the major constraints to animal health and production [21, 22]. There is therefore urgent need to generate and use up-to-date AT prevalence data to refine Uganda AT control priority maps. This is particularly important for Karamoja region where such studies were not previously possible due to insecurity associated with cattle rustling. We therefore carried out this study to generate up-to-date cattle and donkey *Trypanosoma* spp. prevalence data and used it to suggest how AT control priority map for Karamoja region can be refined. In addition, we interviewed animal health providers and key farmers so as to explore the main constraints to livestock health and production in this region.

## Methods

### Study area description

**Fig. 2:**
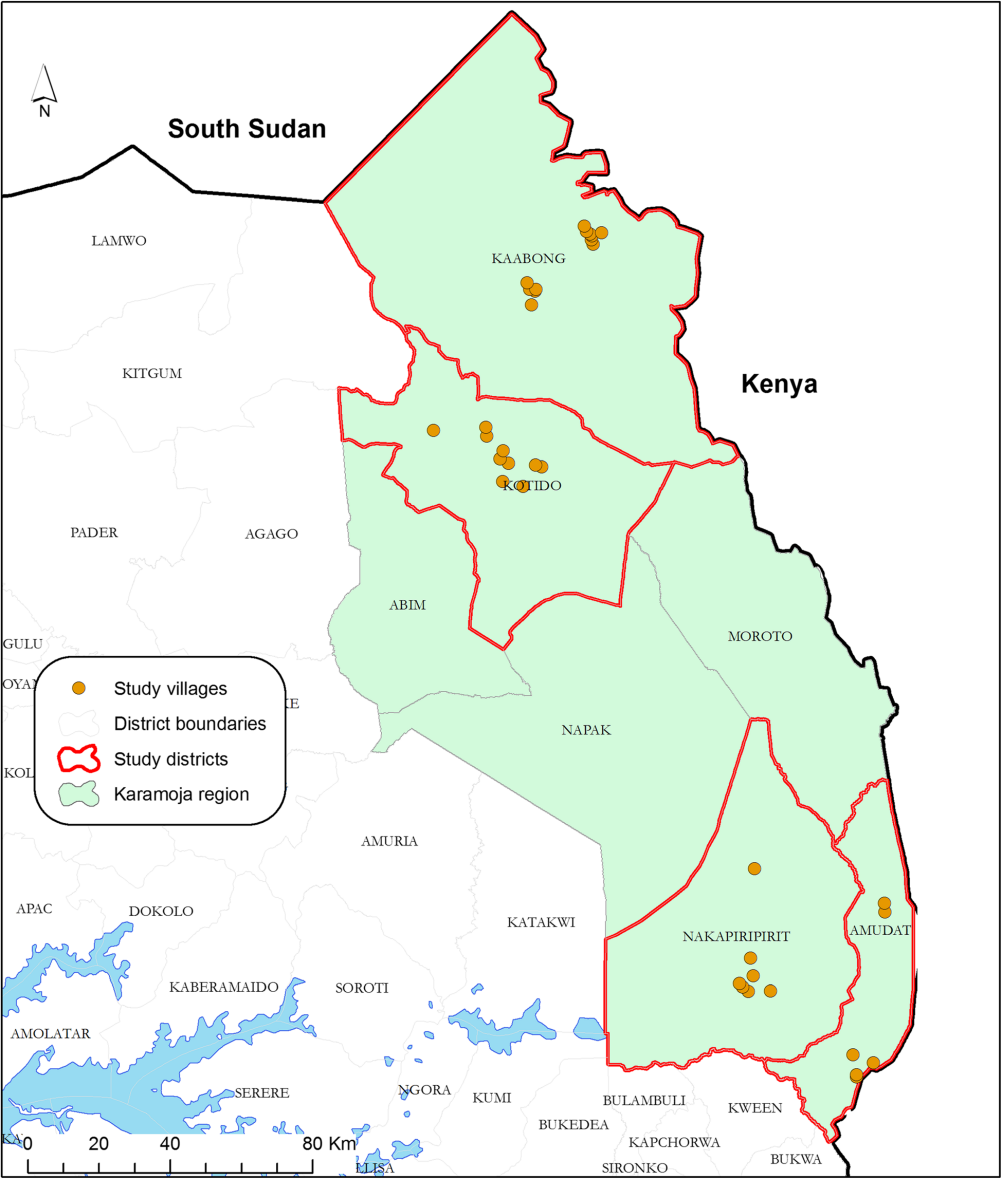
Study area. Seven Karamoja sub-region districts with 4 of the study districts highlighted (red borders)

Karamoja region is a largely remote area in north-eastern Uganda covering about 25,000 km^2^ of land extending over 7 districts between 33 and 35 East and latitude 1–4 North. It is composed of seven districts namely; Kaabong, Abim, Kotido, Moroto, Napak, Nakapiripirit and Amudat. The road network is at its development stage leaving the commonest local means of transport to be footpath trekking by the Karamojong pastoralists and their livestock. Camels and donkeys provide not only animal proteins to the Karamojong but also the most needed means of transport for their agricultural products and livestock production inputs. For this reason, Karamoja region is the only home of the 32,000 camels in Uganda and about 90% (0.134 /0.15 millions) of all the donkey population [23, 24]. This study was carried out in 8 of the 31 sub-counties of Kaabong, Kotido, Nakapiripirit and Amudat districts (Fig. 2). The 8 study sub-counties contained an estimated 75,250 head of cattle distributed within 149 cattle-containing villages (4 target districts): an average of 250 cattle per village. In addition, there were 111,000 head of donkeys; an average of 2 donkeys per village [23].

The climate of the region is semi-arid with a characteristic uni-modal rainfall pattern. The region receives average rainfall of 745 mm (varies widely from 600 mm in the north to 1000 mm in the southern and western parts). The rainy season spans from April to September with scanty rains in June; a main peak in July/August and a minor peak in May. The rest of the months present with an extended dry season (6.5 months) with characteristic high temperatures ranging from 28 to 32.5 °C and an average minimum of 18 °C [25, 26].

Karamoja region is divided into pastoral, agro-pastoral and agricultural livelihood zones depending on the degree of aridity and dependence on livestock. The pastoral region is largely semi-arid and entirely dependent on livestock. It extends from the Kenyan boarder and runs southwards through large parts of Kaabong, Moroto and Amudat districts. This zone experiences prolonged dry seasons and very erratic rainfall. As a result, pastoralists have to move livestock from this zone to other zones for most parts of the prolonged dry season. The agro-pastoral zone extends from the border with South Sudan and covers central parts of Kotido, Kaabong, Moroto, Napak, Amudat and Nakapiripirit districts. This zone receives an average annual rainfall of 500 –800 mm and is largely dependent on livestock and rain-fed crop production. The agricultural zone, the the smallest of the three, covers the western side of Karamoja. The zone supports most tropical food crops because of fertile soils and an average annual rainfall of 700–1000 mm [25, 26].

### Study design and sampling methods

Three stage cluster sampling was used to randomly select study districts, parishes and villages. The initial step involved selection of 4 districts from 7. The second and third stages of sampling included selection of 16 (from 164) and 35 (from 149) parishes and villages respectively. The sampling frame (list of all villages) was obtained from COCTU. With the communal livestock obtaining in Karamoja region, or elsewhere for that matter, AAT tends to cluster at primary cattle grazing units which are villages. A village was therefore taken as the epidemiological unit.

### Sample size estimation

Cluster sampling methodology [27] was implemented in *C Survey* version 2.0 [28] to determine the minimum number of villages (clusters) needed for this survey. In so doing, the following parameters were used; − anticipated prevalence of 5.2% [21], the precision of the sample estimate (one half-length of the 95% confidence interval) of 3 percentage points and an intracluster correlation coefficient-ICC (degree of homogeneity among cattle within the study villages) of 0.15 [10, 20]. Thirty-five (35) clusters (55 cattle each) were required to estimate the apparent prevalence with the set precision. All the Seventy-one (71) donkeys that were presented in the 35 sampled villages were bled.

### Key informant’ and kraal leaders’ interviews

Semi-structured interviews were administered to 21 key informants in order to understand the major constraints to livestock production and health. In addition, 20 focus group discussions were completed with kraal leaders. We selected key informants from key animal health workers in the region. These included community based animal health workers-CBAHWs (*n* = 7), District veterinary officers, heads of production and marketing department (*n* = 5), animal production officers (*n* = 2) and Veterinary officers (*n* = 7). Key informants were employed either by local government departments, non-governmental organisations and intergovernmental organisations (e.g. Food and Agriculture Organization of the United Nations-FAO) operating in Karamoja region. All the interviews were conducted during May 2016.

Livestock are kept in communal herds during the day and in large protected kraals at night. The farmer hierarchy and roles are well structured with the young Karamojong males (5–25 years) in charge of cattle grazing during the day. They return the cattle to communal kraals at night for protection from cattle rustlers as well as for milking. We interviewed kraal leaders (experienced and communally respected farmers) in focus groups (*n* = 10–15) so as to gain an insight of what this category of farmers finds to be the major constraints to livestock production in the region. We also probed them to get an insight of what they find to be the cost of dealing with different animal health problems.

### Key informant and kraal leader interview guides

We asked key informants to enumerate the commonest livestock diseases they often encountered during veterinary practice in their order of importance. They were also asked to explain the methods and cost of dealing with the stated diseases. In addition, we asked them to indicate who often treated (prophylactic and therapeutic) livestock and the cost of their professional fees for each of the diseases. In order to get the key informants’ views about the topical issues under the study, they were given opportunity to ask questions and to freely express such views at the end of each interview. These similar questions were paraphrased in Ngakarimajong (Karamoja local language) in a way understandable by kraal leaders and administered to them by a local Ngakarimajong speaking veterinarian. Kraal leaders’ responses were triangulated with those of key informants. All interview guides were pre-tested and improved to fit their intended purpose before use.

### Individual animal sampling procedure

Given the required sample size (*n* = 55 cattle per village × 35 villages =1925 head of cattle) and an average of 250 animals per village, every 4th presented animal was sampled. On the other hand, all presented donkeys (*n* = 71) were sampled. A temporary paint mark was applied to every sampled animal in order to avoid accidental double sampling. The mean number of cattle and donkeys per village was 252 and 2 respectively. Blood samples were taken from randomly selected cattle (*n* = 2030) and donkeys (n = 71) from 35 villages in the four districts of Karamoja region. Almost all the cattle (99.9%) were of the short horn East African zebu breed (Table 1).

**Table 1:** Description of the cattle and donkey populations

Population attributes	Cattle [N (%)]	Donkeys [N (%)]
A) Age		
0–12 months	276 (13.6)	5 (7.0)
13-24 months	341 (16.8)	10 (14.1)
25-36 months	316 (15.6)	7 (9.9)
>36 months	1097 (54.0)	49 (69.0)
B) Sex		
Female	1273 (62.7)	49 (69.0)
Male	621 (30.6)	22 (31.0)
Neutered	136 (6.7)	0 (0)
C) Breed		
Short horn East African Zebu	2028 (99.9)	N/A
Friesian	2 (0.1)	N/A
*Equus africanus asinus*	N/A	71 (100)

### Cattle and donkey blood sample collection

Middle ear venepuncture was done on each of the sampled animals and 125 μl of blood collected into capillary tubes. The samples were then transferred onto Flinders Technology Associates (FTA) MiniCards (GE Healthcare, Chalfont Station road, UK) using capillary tubes as previously described [29, 30]. The samples were air-dried and sequentially labelled. A summary data collection form (district, county, sub county, parish, village name, coordinates, animal species, age, sex and breed) was completed at each sampling site. Samples were then packed in foil pouches (GE Healthcare) with a silica gel desiccant (Sigma Aldrich, Co., Life sciences, USA) and transported to Makerere university for analysis.

### DNA extraction and *Trypanosoma* spp. detection

DNA was extracted and eluted in Chelex®100 resin (Sigma Aldrich) from five 3 mm FTA sample discs as previously described [29, 31]. Briefly, five discs were punched out from each of the individual samples using a Harris 3.0-mm MicroPunch (GE Healthcare) and discharged into 1.5 ml Eppendorf tubes. The MicroPunch cutting edge was decontaminated after each sample by punching out twice the number of discs from unused filter paper. The sample or negative control discs were incubated twice, each time for 15 min in 1.0 ml FTA Purification Reagent (GE Healthcare). This was followed by two rinses of 15 min in 1.0 ml of 1×TE buffer (10 mM Tris–HC_l_, 0.1 mM EDTA, pH 8.0). Incubation was then done with agitation at room temperature. Thereafter, the discs were dried at 37 °C for 30 min in an incubator (Heldoph, Schwabach, Germany) or were left to dry at room temperature overnight. DNA was eluted from the discs in 100 μl of 5% *w*/*v* Chelex/RNA and DNase-free water at 90 °C in a thermocycler (My cycler, Bio-Rad, USA). Eluted DNA samples were kept at −20 °C for long term PCR analyses or 4 °C if they were to be analysed within a week of extraction.

### Trypanosome DNA detection

Eluted DNA samples were screened for different trypanosome species using a single pair of internal transcribed spacer 1 (ITS1) CF/BR primers and thermo-cycling conditions as previously described [32]. The ITS1- PCR was carried out in 25 μl reactions. Each reaction contained 5 μl of the test sample, negative or positive control, 1×–reaction buffer (670 mM Tris–HC_l_ pH 8.8, 166 μM (NH4)_2_SO_4_, 4.5% Triton X-100, 2 mg/ml gelatin) (Bioline, Humber Road, London, UK), 2.5 mM MgCl_2_, 200 μM of each dNTP, 5 μM each of the CF and BR primers, 0.5 U of *BioTaq*DNA polymerase (Bioline, Humber Road, London, UK), and 15.2 μl of RNA and DNase-free water.

The multiplex serum-resistance associated gene (SRA)-PCR was carried out on each of the samples from which a 480 bp fragment was detected by ITS1-PCR. The multiplex SRA-based PCR simultaneously detects 324, 669 and 800 bp fragments of the glycosylphosphatidylinositol-phospholipase C (GPI-PLC), SRA and variable surface glycoprotein (VSG) genes respectively. Serum-resistance associated gene is specific to *T. b. rhodesiense* while GPI-PLC is a Trypanozoon specific marker. Glycosylphosphatidylinositol-phospholipase C is included as a sample DNA quality control. A sample would be considered positive for *T. b. rhodesiense* only if SRA gene band size were detected; otherwise its absence, but with presence of the GPI-PLC or VSG bands would imply presence of other Trypanozoon trypanosomes [*T. B. brucei* or *T. evansi*]. The samples from which VSG and GPI-PLC bands were amplified were subjected to *T. evansi* specific PCR [33, 34] so as to determine whether they were positive for *T. B. brucei* or *T. evansi*. Samples were not checked for presence of *T. b. gambiense* because *T. b. gambiense* is known to be limited to north-western Uganda districts. Multiplex PCR was carried out in 25 μl reactions using primers and conditions as previously described [35].

All samples from which a ≥ 600 bp fragment was amplified on ITS1-PCR were screened for *T. congolense* savannah / kilifi and forest using TC1/2, TK1/2 and TF1/2 primer sets. This was done in order to determine the commonest *T. congolense* genotype(s) circulating in Karamoja region. The congolense strain specific PCRs were completed in 25 μl reactions as previously described [36]. Each reaction contained 5 μl of the test sample, negative or positive control, 1×–reaction buffer (670 mM Tris–HCl pH 8.8, 166 μM (NH4)_2_SO_4_, 4.5% Triton X-100, 2 mg/ml gelatin) (Bioline, Humber Road, London, UK), 0.75 mM MgCl_2_, 200 μM of each dNTP, 12.5 μM each of the TC1/2, TK1/2, TF1/2primers, 1 U of BioTaqDNA polymerase and 13.05 μl of RNase-free water.

Donkey and cattle samples from which VSG and GPI-PLC bands were amplified by multiplex SRA-PCR were subjected to to *T. evansi* specific PCR using TeRo-Tat920F/ TeRo-Tat1070R primers that amplify a 151 bp fragment of the *T. evansi* RoTat 1.2 VSG gene. The *T. evansi* specific PCR was completed in 25 μl reactions using thermal cycling conditions as previously described [33, 34].

PCR products for the six sets of PCRs were separated in 1.5% agarose (Bio Tolls Inc. Japan), stained in ethidium bromide (Sigma Aldrich, Co., Life sciences, USA) and visualised on an ultraviolet transilluminator (Wagtech International, Thatcham, UK) for fragment size determination.

### Statistical analyses

Key informants’ and kraal leaders’ responses were coded and analysed in Microsoft Office Excel 2016 by coding and memo writing methods [37, 38]. Trypanosome prevalence and their corresponding confidence intervals (CIs) were estimated using generalised estimating equation models with binary outcome and logit link function. Robust sandwich standard errors accounted for correlation within villages. The analysis was performed in R statistical software (geepack-package) version 3.3.1. ArcMap v10.3 (spatial analyst extension) was used to map prevalence estimates in different villages.

## Results

### Livestock health constraints in Karamoja region

Tick-borne diseases namely; − East Coast Fever (ECF), Anaplasmosis, Cowdriosis and Babesiosis were ranked by 75% (14/21) of the key informants as the most commonly encountered diseases during their veterinary practice. A quarter (5/21) of this group also ranked AAT as the second most commonly encountered livestock disease. African animal trypanosomiasis was reported to be mostly encountered in Kotido and Amudat districts. Key informants reported Foot and Mouth Disease (FMD), Contagious Bovine Pleuropneumonia (CBPP), Caprine Pleuropneumonia (CCPP), Peste des Petits Ruminants (PPR), Helminthiasis, Lumpy Skin Disease (LSD), Foot Rot and Brucellosis as the other important diseases in descending order of importance.

On the other hand, livestock keepers in 8 of the 20 focus group discussions intimated that TBDs were the most important causes of losses in their livestock. The rest of the farmers split in equal halves (of 6/20) indicated that either AAT or CBPP were the most important constraints to livestock production. These responses were fairly uniform in the farmers’ groups within the four study districts. Just like the animal health providers, farmers reported LSD, FMD, Helminthiasis, foot rot, black quarter and Brucellosis as some of the other livestock diseases that constrain livestock production in the region.

### Livestock disease control methods

Key informants indicated that farmers controlled livestock diseases using three main strategies. These included vaccination (for example against FMD, LSD and CBPP), insecticide / acaricide application (TBDs and AAT) and non-characterised herbal extracts (ticks and tsetse). Livestock production inputs including vaccines, acaricides, insecticides were largely provided by government (district veterinary offices), non-governmental organisations or FAO and least by farmers themselves. All key informants indicated that chemotherapeutic and chemoprophylactic management of the most common livestock diseases was largely (95% of the time) done by CBAHWs and farmers. The majority (98.9%) of the kraal leaders’ responses on ways of dealing with major livestock diseases were similar to those of key informants.

### Livestock diseases associated with highest financial losses in Karamoja region

Whereas this study was not designed to provide an extensive financial or economic analysis of different causes of losses to the livestock sector in Karamoja region, we sought kraal leaders’ and key informants’ experiences of the major causes of financial / economic losses to the sector. TBDs, AAT, CBBP, CCPP, PPR and FMD were reported to be the most important livestock diseases in descending order. The main causes of losses to the livestock industry (albeit not quantified) were reported to be due to mortalities, morbidities, quarantine (FMD, CBPP, CCPP & PPR) and costs of prevention and treatment of these diseases. Tick-borne disease  treatment was reported to be associated with the highest financial losses (UGX 2,5000–75,000 [USD 7–21] depending on the individual TBD. On the other hand, AAT case management cost UGX 3000–4000 [USD 0.8–1.1] if diminazene or Isometamidium was administered respectively (Table 2). Professional fees were not included in these estimates since majority of them were administered by farmers or CBAHWs. The latter were only paid the cost of drugs if they helped farmers administer treatments.

**Table 2:** Relative cost of managing main livestock diseases in Karamoja region

Disease	Karamoja region cattle	Number affected annually^c^	Direct Cost (UGX) of managing a case	Total Direct costs (billion UGX)	Total Indirect costs^a^ (billion UGX)	Total costs	
						Total Costs (billion UGX)	Total (million USD)
Anaplasmosis, Babesiosis, Cowdriosis	2,600,000	468,000.0	25,000.0	11.7	9.1	20.8	6.1
ECF		468,000.0	75,000.0	35.1	27.4	62.5	18.3
Nagana		452,400.0^d^	3-4000^b^	5.4	4.2	9.6	2.8
Total	2,600,000	1,388,400.0		52.2	40.7	92.9	27.2

Uganda shillings (*UGX*), United States Dollars (*USD*)

Costs were triangulated from responses of 20 kraal leaders’ focus groups as well as 21 individual key informants. Abroad brush analysis was undertaken using already published literature to generate indicative costs of dealing with AAT and TBDs

^a^ Direct cost of managing vector-borne diseases like TBDs and AAT have previously been noted to be ~77.6% of the direct costs [9, 41]

^b^ 4 Curative (diminazene) treatments per year (total UGX 12,000) or 3 prophylactic (isometamidium) treatments per year (UGX 12, 000)

^c^ Tick-borne diseases (ECF, Anaplasmosis, Babesiosis) have been reported recently to have an incidence rate of 18% in the Karamoja region [22]

^d^ Represents 17.4% of all cattle in Karamoja region; the proportion that this study found to be infected with at least a single economically important trypanosome

Key informants reported the average cost of treating a single ECF case as UGX 75,000 [USD 21] and that of other TBDs to be UGX 25,000 [USD 7]. In addition, they reported the cost of treating AAT case to be UGX 3000–4000 [USD 0.8–1.1] depending on whether curative (diminazene) or prophylactic (Isometamidium) trypanocidal treatments were administered. Since farmers treated sick animals in almost all cases, it was hard to quantify the cost of professional fees for managing most important livestock diseases in Karamoja region. In addition, CBAHWs charged non-monetary items including milk and other food items that were had to express in monetary terms since their quantities varied widely.

### Trypanosome species prevalence in cattle and donkeys in Karamoja region

Sixteen percent (95% CI; 12.4–21.1%) of the cattle were infected with at least one of the three detected trypanosome species namely *T. vivax*, *T. congolense savannah (T. congolense)* and *T. brucei brucei (T. brucei).* Twenty-two cattle had mixed trypanosome infections. The most common co-infections observed were *T. vivax* and *T. congolense* (17/2030), *T. vivax* and *T. brucei* (3/2030). Only one cow was co-infected with *T. congolense* and *T. brucei)*. *T. brucei* was the least prevalent (0.9%, 95% CI: 0.5–1.7%) trypanosome species*. T. congolense savannah* was the only circulating *T. congolense* strain in the cattle sampled (Table 3).

**Table 3:** Prevalence of T*rypanosoma* spp. in cattle and donkeys in Karamoja region (May 2016)

Trypanosoma spp.	Cattle (n = 2030)			Donkeys (n = 71)		
	Positive	% prevalence (95%CI)	% Herd prevalence^a^	Positive	% prevalence (95%CI)	% Herd prevalence^a^
Overall	331	16.3 (12.4–21.1)	85.7	23	32.4 (20.2–47.6)	57.1
*T. vivax*	246	12.1 (9.0–16.1)	80.0	4	5.6 (2.2–13.6)	21.4
T. congolense	90	4.4 (3.0–6.5)	68.6	21	29.6 (18.6–43.6)	57.1
*T. brucei* s.l.	17	0.9 (0.5–1.7)	25.7	0	0.0	0.0

^a^Due to the local farming systems, all animals within a village are considered as a herd

Forty percent (14/35) of the villages sampled kept donkeys. The donkey to cattle proportion was about 1:100. Four of the seventy-one donkeys (5.6%, 95% CI: 2.2–13.9%) were positive for *T. vivax* while 21 (29.6%, 95% CI: 18.6–43.6%) were positive for *T. congolense*. Only 2 donkeys were co-infected with *T. vivax* and *T. congolense*. The overall prevalence of different trypanosome species in donkeys tested was 32.4% (95% CI: 20.2–47.6%) (Table 3). Donkey herd as well as the district level distribution of trypanosome infections were highly clustered in Kotido district (Table 4).

**Table 4:** Donkey *Trypanosoma* spp. herd level prevalence in Karamoja region

Village	District	Sampled (n)	Percentage prevalence			
			T. spp.	*T. vivax*	*T. brucei*	*T. congolense*
Nasinyon	Kotido	16.0	56.3	6.3	0.0	50.0
Nadomeo	Kotido	6.0	50.0	0.0	0.0	50.0
Nakumoit	Kotido	4.0	25.0	25.0	0.0	25.0
Lokiding	Kotido	6.0	16.7	0.0	0.0	16.7
Lotanyat	Kotido	3.0	66.7	0.0	0.0	66.7
Kalogwel	Kotido	5.0	40.0	20.0	0.0	40.0
Kanameriongor	Kotido	8.0	37.5	25.0	0.0	25.0
Locheger East	Kaabong	2.0	0.0	0.0	0.0	0.0
Morudikae	Kaabong	4.0	0.0	0.0	0.0	0.0
Nariwore	Kaabong	2.0	0.0	0.0	0.0	0.0
Lochoto	Kaabong	2.0	0.0	0.0	0.0	0.0
Lolelia centre	Kaabong	4.0	50.0	0.0	0.0	50.0
Lokadangan	Nakapiripirit	4.0	0.0	0.0	0.0	0.0
Moruarengan	Amudat	5.0	0.0	0.0	0.0	0.0
Totals	14	71.0	32.4	5.6	0.0	29.6

*T. spp. Trypanosoma* spp. namely; −*T congolense, T vivax* and *T brucei s.l*

### Spatial distribution of different bovine trypanosome species

The spatial distribution of *Trypanosoma* spp. in cattle and donkeys tested was highly clustered at both village and district levels. Six out of the 35 villages recorded no trypanosomes in cattle sampled. The highest prevalence of trypanosomes in cattle was recorded in Kotido district while the highest cattle herd prevalence of 65% was recorded in Kotarukot village in Kaabong district (Table 5). The ICC of *Trypanosoma* spp. was 0.14. *T. vivax* was the most common (12.1%, 95% CI; 9.0–16.1%) trypanosome detected in all cattle sampled. In addition*,* 80% of all the 35 sampled cattle herds were positive for *T. vivax*. *T. congolense* and *T. brucei* were detected in 69% and 26% of all the 35 cattle herds sampled respectively. None of the *T. brucei s.l.* DNA positive samples were positive for the SRA gene indicating that they were all *T. b. brucei* positive.

**Table 5:** Bovine *Trypanosoma* spp. herd level prevalence in Karamoja region

Village	District	Sampled (n)	*Percentage trypanosome prevalence*			
			T. spp	*T. vivax*	*T. brucei*	*T. congolense*
Loputuk	Kotido	56	30.4	19.6	0.0	10.7
Nasinyon	Kotido	56	23.2	14.3	0.0	8.9
Nadomeo	Kotido	62	33.9	19.4	1.6	12.9
Nakumoit	Kotido	56	12.5	8.9	1.8	1.8
Namukur	Kotido	56	33.9	16.1	7.1	10.7
Lokiding	Kotido	56	19.6	7.1	5.4	7.1
Lotanyat	Kotido	60	35.0	16.7	0.0	18.3
Poet	Kotido	56	10.7	10.7	0.0	0.0
Kalogwel	Kotido	60	28.3	21.7	0.0	6.7
Kanameriongor	Kotido	62	17.7	11.3	0.0	6.5
Kanakuruk	Kaabong	56	1.8	0.0	0.0	1.8
Locheger East	Kaabong	50	2.0	2.0	0.0	0.0
Locheger West	Kaabong	50	2.0	0.0	0.0	2.0
Loputuk	Kaabong	56	1.8	1.8	0.0	0.0
Morudikae	Kaabong	56	0.0	0.0	0.0	0.0
Napeichokei	Kaabong	50	2.0	2.0	0.0	0.0
Nariwore	Kaabong	56	10.7	8.9	0.0	1.8
Kotarukot	Kaabong	60	65.0	53.3	1.7	10.0
Lobalangit	Kaabong	56	16.1	14.3	0.0	1.8
Lochoto	Kaabong	56	25.0	17.9	1.8	5.4
Lolelia Centre	Kaabong	60	13.3	10.0	0.0	3.3
Nakwakwa	Kaabong	56	16.1	16.1	0.0	0.0
Aoyalira	Nakapiripirit	60	0.0	0.0	0.0	0.0
Apeicorait	Nakapiripirit	60	0.0	0.0	0.0	0.0
Lokitela	Nakapiripirit	60	0.0	0.0	0.0	0.0
Lokadangan	Nakapiripirit	60	10.0	8.3	0.0	1.7
Nakiloro	Nakapiripirit	60	5.0	5.0	0.0	0.0
Naabore-B	Nakapiripirit	46	30.4	23.9	4.3	2.2
Arengesiep	Nakapiripirit	60	0.0	0.0	0.0	0.0
Abongai	Amudat	82	31.7	18.3	3.7	9.8
Korenyang	Amudat	60	20.0	11.7	0.0	8.3
Moruarengan	Amudat	60	15.0	13.3	0.0	1.7
Morumodo	Amudat	60	35.0	15.0	3.3	16.7
Angarab	Amudat	60	30.0	30.0	0.0	0.0
Tingas	Amudat	60	18.3	18.3	0.0	0.0
Totals		2030	16.3	12.1	0.9	4.4

*T. spp. Trypanosoma* spp. namely; −*T. congolense, T. vivax* and *T. brucei s.l*

## Discussion

Livestock health workers reported in this study, just like in previous studies [22, 39], that TBDs (ECF, Anaplasmosis, Cowdriosis and Babesiosis), AAT and CBPP are the top three important livestock health constraints in Karamoja region. East coast fever has recently been reported to be non-endemically stable within Karamoja region explaining why the disease was reported to be associated with highest incidence and mortalities in the region [22, 39]. This study indeed confirmed that AAT is a major cause of losses to the livestock sector since 17.4% of the cattle sampled were positive for economically important trypanosome DNA.

Byaruhanga and colleagues reported that livestock which suffer from either TBDs or AAT are likely to die (mean case fatality rates; 67–90%) [22]. On the other hand, this study just like previous studies [22, 39, 40], found out that animal health workers and farmers’ have high level of understanding of TBDs and AAT clinical signs, right control and treatment methods. Failure to effectively control and treat TBDs and AAT much as the different actors have sufficient knowledge to do so is most likely to be as a result of farmers and CBAHWs treating over 95% of the sick animals. As such, farmers and key informants indicated that veterinarians only treat 5% of the sick animals. Low farmers’ willingness to pay veterinarians to treat their cattle is a likely reason why most animals are treated by farmers and CBAHWs resulting into infective treatment/control of TBDs and AAT.

Non-governmental and inter-governmental organisations (mainly FAO) working in Karamoja region train CBAHWs (who are largely farmers with not much formal training) to equip them with basic knowledge about livestock disease clinical signs and their control methods. This level of training does not seem to be sufficient to help CBAHWs be able to administer right treatment regimens for different livestock diseases. There is therefore need to retrain them about effective treatment regimens of TBDs and AAT as well as establish and maintain a regulatory framework for the involvement of CBAHWs in animal health.

Given that recent studies have indicated that the indirect costs associated with tsetse and tick-borne disease management are up to 77.6% of direct costs [9, 41], the indirect costs associated with AAT and TBD management by farmers in Karamoja region is about UGX 40.7 billion (US$ 11.9 million). These costs would be higher if adjusted to include costs due to loss in production, mortalities and vector control. Even at indicative costs (before they are refined in more exhaustive financial and economic studies) level, the total costs of dealing with AAT and TBDs (UGX 93 billion, US$ 27.2 million) are about 7 times the annual government support to the department of production and marketing (UGX 14.4 billion; ~US$ 4.32 million) in the whole Karamoja region. This explains why farmers and livestock health professionals in this region unmistakably ranked TBDs and AAT as the two most important constraints to livestock health.

**Fig. 3:**
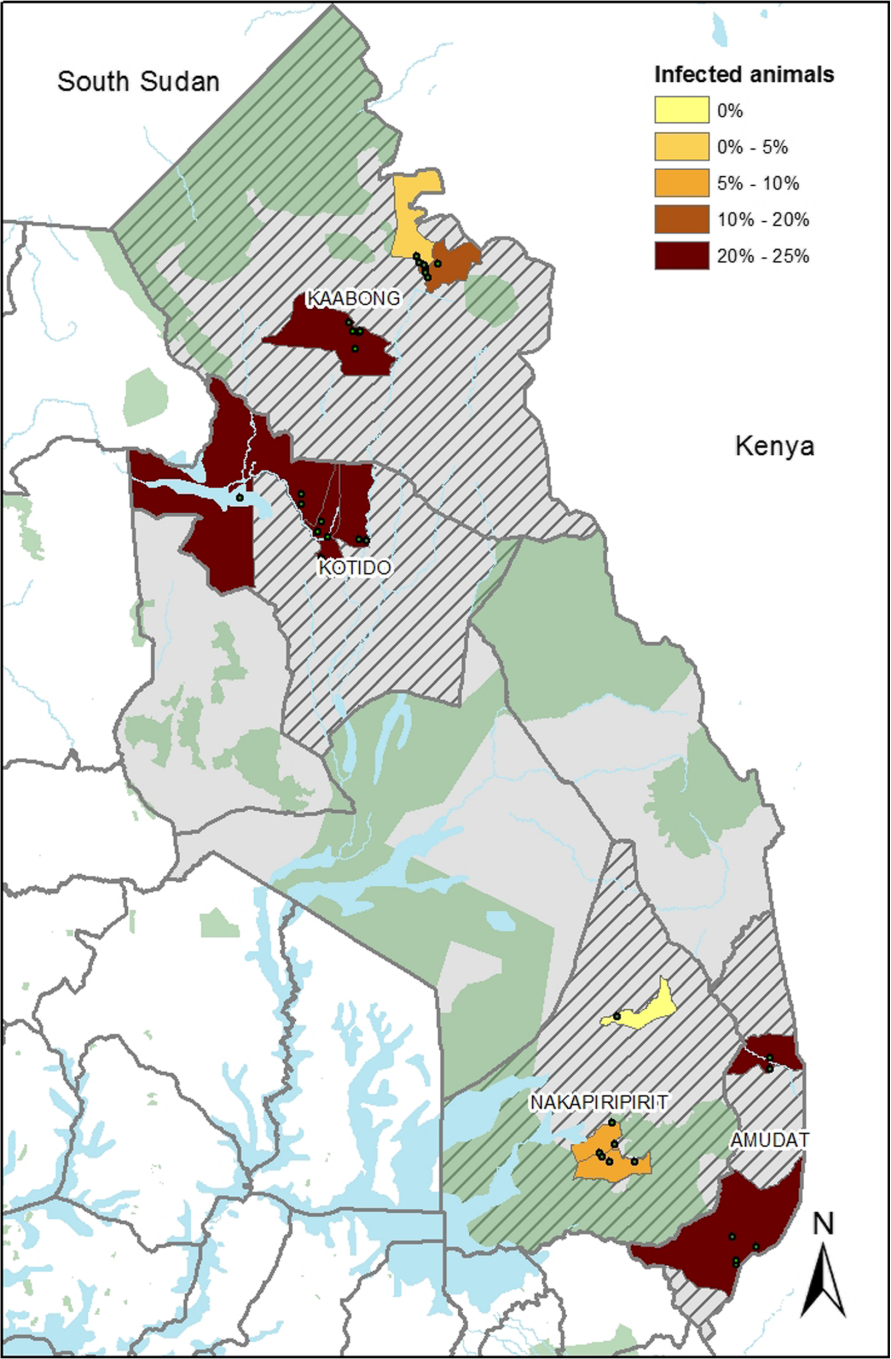
Proportion of animals infected by any *Trypanosoma* spp. Data were aggregated at parish level because selected villages were within the interpolation distance and could not otherwise give enough spatial resolution

A previous small trypanosomiasis survey (*n* = 196) in Kotido district [21] reported an overall prevalence of 5.2% much lower than is reported in this study. However, the trypanosomes species detected during the previous survey are largely similar to those detected in this study. The large difference (12.5%) in the overall prevalence of bovine trypanosome species previously reported in Kotido district [21] and this study is likely to be due to the differences in the sizes (number of cattle sampled, methods of sampling and the districts included) of both surveys as well as the time differences. To our knowledge, this is the first large (covering cattle and donkeys in 4/7 districts of Karamoja region) AAT survey in Karamoja region. This survey indicates that AAT is a major constraint to livestock production in Karamoja region (Fig. 3). This implies that there is need to re-define Karamoja region as a high priority area for trypanosomiasis control.

*T. vivax* undergoes a very short life cycle in the proboscis of tsetse flies [42] and is associated with rapid build-up of parasitaemia in its mammalian hosts [10, 16]. These two features make it very easy to transmit cyclically. *T. vivax* is also known to be mechanically transmitted by a range of hematophagous flies making it appear in areas beyond known tsetse belts [16]. This partly explains why *T. vivax* was the most prevalent trypanosome species detected in this study just like in recent studies in other AAT endemic regions of Uganda [10, 20].

*T. congolense savannah* and *T. b. brucei* were detected in low overall and herd prevalence in the four districts of Karamoja region. *T. brucei s.l.* and *T*. *congolense savannah* low herd and overall prevalence have been reported elsewhere in AAT endemic regions of Uganda [10, 20]. *T. congolense savannah* is highly pathogenic to cattle which partly explains its low prevalence in most routine molecular epidemiological studies involving apparently healthy cattle [10]. None of the *T. brucei s.l. DNA* positive samples were positive for the serum resistance antigen (SRA) gene indicating that none of them was human infective (*T. b. rhodesiense*). *T. b. rhodesiense* infections can be very focal in the cattle and wild life reservoir especially in non-epidemic states [20]. The current indication of no risk of sleeping sickness in this region needs to be confirmed by large-scale surveys involving all districts in the Karamoja region.

**Fig. 4:**
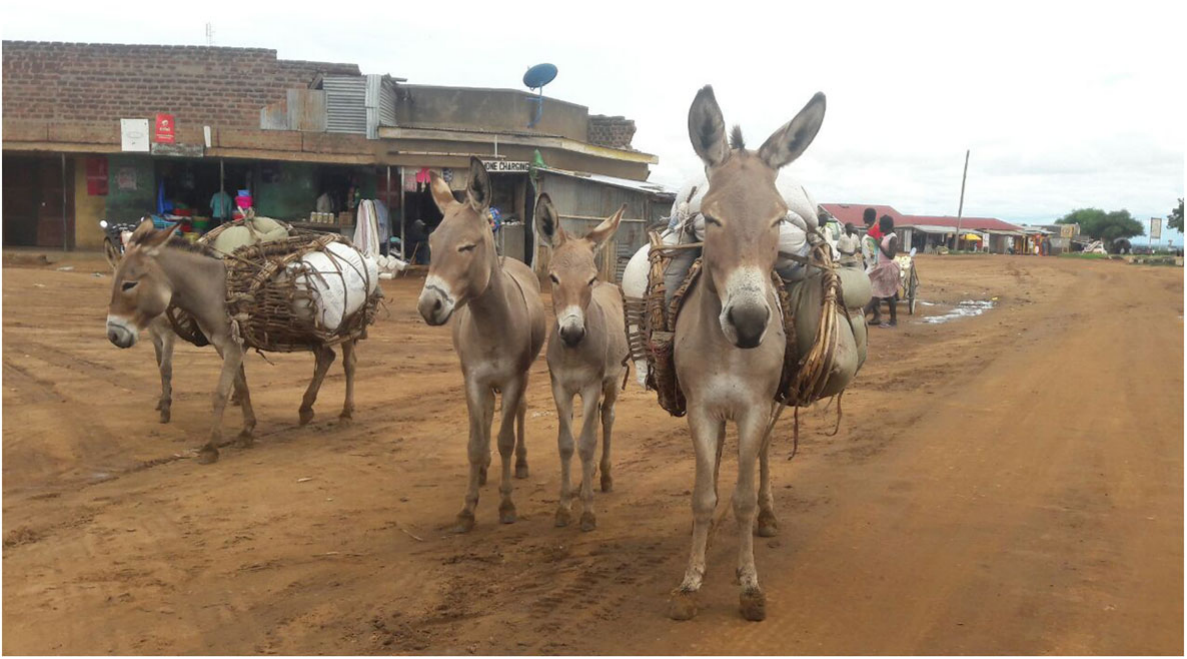
Donkeys used as the main form of transport for agricultural products and inputs in Kotido district, northern Karamoja (Credit: Dennis Muhanguzi)

Donkeys and camels provide animal proteins as well as transport of agricultural products and inputs [Fig. 4]. There is need to reflect this key role of donkeys and camels in providing for their health, in this case looking at the most important trypanosomes that constrain donkey health, production and management. *T. congolense savannah* and *T. vivax* were detected in high proportions in donkeys at herd and district levels. Both *Trypanosoma* spp. are reported to cause chronic AAT in donkeys [43, 44]. Given that the two trypanosome species were the most prevalence in cattle as well, there is need to study the role of donkeys in the epidemiology of other livestock AAT.

Bovine and equine trypanosome species were highly clustered at both village and district levels (Fig. 4). This kind of clustering pattern has recently been reported in the AAT endemic regions of south-eastern Uganda [10, 20]. Clustered distribution of trypanosome infections can be attributed to different factors. These include but are likely not limited to; −level of challenge and differences in livestock management practices practiced by farmers in different villages and districts [10, 19, 20].

## Conclusion

Livestock production remains the mainstay of the Karamajong livelihoods. On the other hand, livestock production potential in this region is limited by endemic livestock diseases including AAT and TBDs. We report here that 16.3% and 32.4% of all cattle and donkeys sampled respectively were infected with different *Trypanosoma* spp. This was in strong agreement with farmers’ and key informants’ observations that AAT is only second to TBDs in constraining livestock production in Karamoja region. In order to improve livestock health and production, it is therefore apparent that government of Uganda needs to invest in livestock health and production programs particularly AAT and TTBD control. Karamoja region AT priority control map should be refined using the new AT prevalence data (arising from this survey and those in future) so as to highlight the region as a high priority region for trypanosomiasis control.

## Abbreviations

AAT: Animal African Trypanosomiasis
AT: African Trypanosomiasis
CBAHWs: Community based animal health workers
CBPP: Contagious Bovine Pleuropneumonia
CCPP: Caprine Pleuropneumonia
CI: Confidence interval
ECF: East Cost Fever
FAO: Food and Agriculture Organization of the United Nations
FMD: Foot and Mouth Disease
FTA: Flinders Technology Associates
GPI-PLC: Glycosylphosphatidylinositol-phospholipase C
HAT: Human African Trypanosomiasis
ICC: Intracluster Correlation Coefficient
ITS1-PCR: Internal transcribed spacer 1- based polymerase chain reaction
LSD: Lumpy Skin Disease
PPR: Peste des petits ruminants
SRA: Serum-resistance associated gene
TBDs: Tick-borne diseases
TTBDs: Ticks and tick-borne diseases
UGX: Uganda shillings
USAID: United States Agency for International Development
USD: United States Dollars
VSG: Variable surface glycoprotein
